# Serial T2-mapping to quantitatively monitor resorption of myocardial edema following acute myocardial infarction

**DOI:** 10.1186/1532-429X-15-S1-P179

**Published:** 2013-01-30

**Authors:** Gunnar Lund, Kai Muellerleile, Peter Bannas, Julia Cuerlis, Dominik Barz, Ulf K Radunski, Christian Stehning, Bernhard Schnackenburg, Karsten Sydow, Gerhard Adam

**Affiliations:** 1 Radiology, University Hospital, Hamburg, Germany; 2 Cardiology, University Hospital, Hamburg, Germany; 3 Philips Research, Hamburg, Germany

## Background

Currently, myocardial edema monitoring after acute myocardial infarction (AMI) is based on visualization of the region with increased signal-intensity on T2-weighted images. T2-mapping is a promising novel cardiac magnetic resonance imaging (CMR) technique to quantitatively assess myocardial edema. The purpose of the study was to quantitatively evaluate resorption of myocardial edema following AMI using T2-mapping.

## Methods

CMR (1.5 Tesla Philips Achieva) was performed in 20 patients within seven days after a reperfused AMI (Baseline) and at one, three and six months follow-up, respectively. A free-breathing, navigator-gated multi-echo sequence was used for T2-mapping. T2-maps were calculated from fifteen echoes using a dedicated plug-in written for OsiriX software. Serial T2 values were assessed using a six-segment model (Figure). Infarcted and remote segments were defined by using information from corresponding late-enhancement images (Figure).

**Figure 1:**
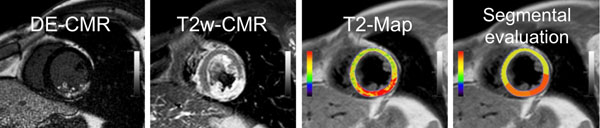
CMR of a patient with acute posterior infarction. The infarct and the myocardial edema is seen on the DE- and the T2w-CMR, respectively. The T2-Map shows increased T2 time in the infarcted area. A segmental evaluation was performed in six segments.

## Results

A significant decrease of the T2 time was found in infarcted segments from baseline to one month follow-up (82±19 vs. 70±7 ms; p<0.05), but not between one and three (70±7 vs. 62±8; p=ns) or three and six (62±8 vs. 62±5 ms; p=ns) month follow-up. Identical T2 times were found in remote segments at baseline, one, three and six month follow-up (61±6 vs. 61±5 vs. 59±7 vs. 58±2 ms; p=ns). The T2infarct/T2remote ratio decreased from baseline to one month follow-up (1.34±0.26 vs. 1.15±0.10; p<0.05) and from one to three month follow-up (1.15±0.10 vs. 1.04±0.06; p=0.05). No significant change was found for the T2infarct/T2remote ratio from three to six month follow-up (1.04±0.06 vs. 1.07±0.09; p=ns).

## Conclusions

Serial T2-mapping enables monitoring of edema resorption following acute myocardial infarction. The magnitude of edema resorption occurs in the first month after AMI and the T2 values normalize within 3 months after AMI. Thereafter, no further T2 time reduction is observed.

## Funding

None

